# Supplementary material for: Sequential screening nominates the Parkinson's disease associated kinase LRRK2 as a regulator of Clathrin-mediated endocytosis
Source: Neurobiol Dis. 2020 Jul;141:104948. doi: 10.1016/j.nbd.2020.104948 (PMC7339134; doi:10.1016/j.nbd.2020.104948)
Supplement: Supplementary file 1 — Supplementary material 1 [file mmc1.pdf]

Supplementary Figure 1.

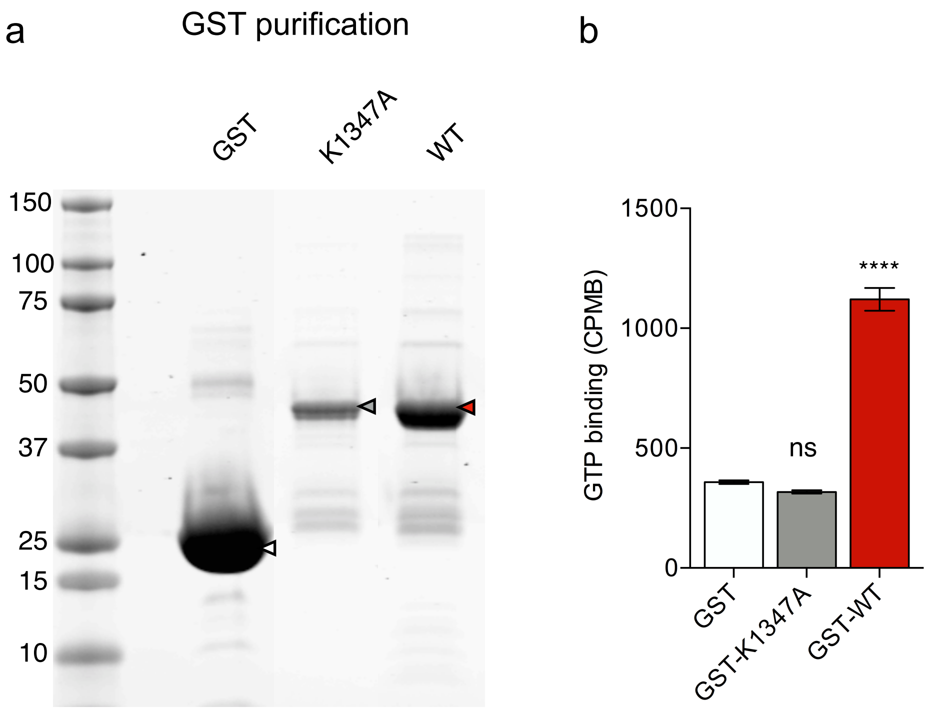

Supplementary Figure 2.

a

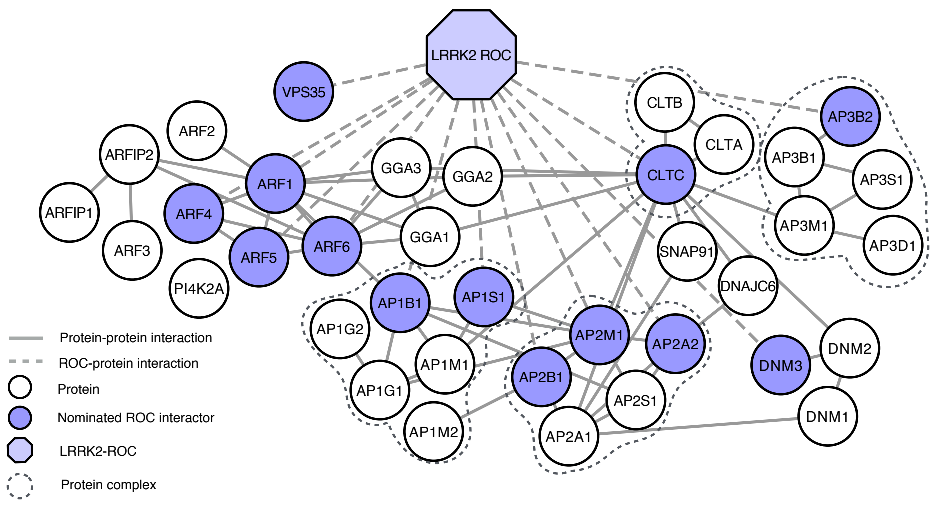

b

Gene	MeanZ	Screen 1 Z	Screen 2 Z	Adj. P
QL	-3.9069	-2.8979	-4.8974	2.8000E-54
CK1a	4.9899	2.7705	7.1299	9.2200E-37
ARHGEF7	-1.5981	-1.0429	-2.1533	1.4800E-17
AP1B1	2.7821	2.1685	3.3190	8.4200E-06
ARF4	2.7109	2.4184	3.0034	9.5900E-06
ARF6	2.6403	2.4203	2.8602	2.4800E-05
AP2A1	4.1338	2.2270	6.0405	0.0002
ARF1	-1.8558	-1.0660	-2.6457	0.0006
AP2A2	1.6937	1.7247	1.6666	0.0009
DNM2	2.7760	1.4091	4.1430	0.0010
AP1M1	3.9483	1.5947	6.3019	0.0031
ARF3	-1.6050	-0.9495	-2.1786	0.0058
AP2M1	2.1563	0.7550	3.5577	0.0305

P < 0.05

GGA2	1.0825	0.6445	1.5205	0.1469
AP3S1	-0.7647	-0.7529	-0.7782	0.2751
AP1M2	1.0052	0.6268	1.3363	0.5082
CLTA	-0.6811	-0.4791	-0.8579	0.6141
GGA1	-0.9354	-1.8144	-0.1663	0.6159
AP1G2	-0.5424	-0.3752	-0.7096	0.7309
CLTB	-1.2799	-1.4830	-1.0768	0.7726
AP3B1	-0.6350	-0.2952	-0.8898	1.0000
GGA3	-0.5631	0.1132	-1.2394	1.0000
AP1S1	-0.4277	-0.6314	-0.2494	1.0000
DNM1	-0.3980	-0.7538	0.0087	1.0000
DNAJC6	-0.3319	-0.9135	0.2497	1.0000
DNM3	-0.3257	-0.4909	-0.2018	1.0000
AP2S1	-0.3014	-1.1190	0.5162	1.0000
CLTC	-0.2952	-0.9897	0.3993	1.0000
AP3D1	-0.0420	0.0679	-0.1382	1.0000
SNAP91	0.2111	-0.0651	0.4873	1.0000
PI4K2A	0.3820	0.3825	0.3815	1.0000
VPS35	0.4090	0.5897	0.2025	1.0000
ARFIP1	0.4159	-0.2041	0.8809	1.0000
ARF5	0.4356	1.1830	-0.3119	1.0000
AP3B2	0.4753	0.0492	0.8482	1.0000
AP3M1	0.5321	-0.3655	1.4296	1.0000
AP1G1	0.6736	1.3136	0.0337	1.0000
ARFIP2	0.7438	0.3163	1.1179	1.0000
AP2B1	1.0545	-0.0139	2.1229	1.0000

c

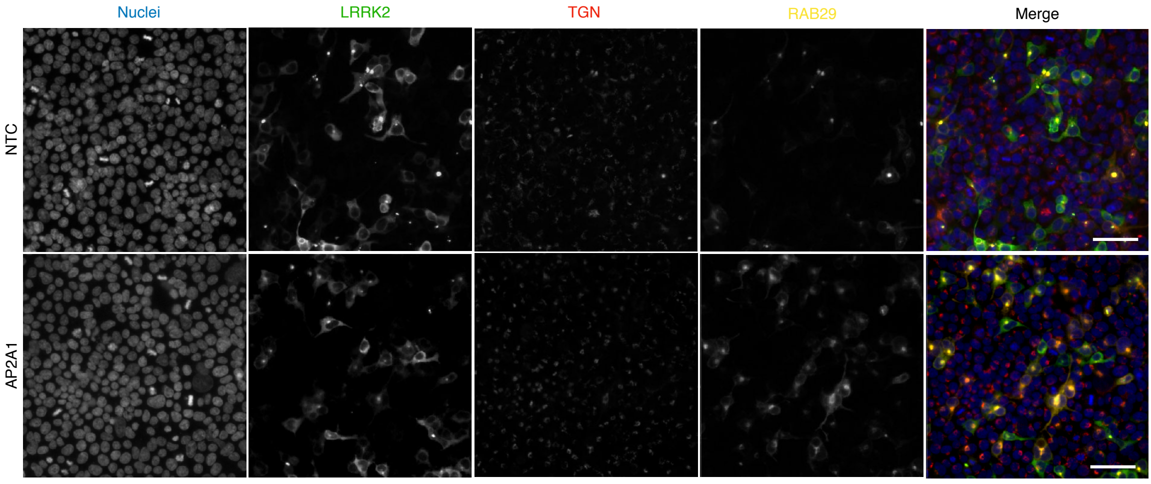

Supplementary table 3.

Accession	MASCOT Score	Mass	Peptide matches	Significant peptide matches	Sequence matches	Significant sequence matches	emPAI	Protein	Gene	Tissue
AP2B1_MOUSE	1955	104516	100	63	49	35	4.26	AP-2 complex subunit beta	AP2B1	Brain
AP2A2_MOUSE	1215	103951	79	41	44	27	2.68	AP-2 complex subunit alpha-2	AP2A2	Brain
AP1B1_MOUSE	1022	103869	73	39	43	30	2.84	AP-1 complex subunit beta-1	AP1B1	Brain
AP2M1_MOUSE	487	49623	47	23	26	16	3.61	AP-2 complex subunit mu	AP2M1	Brain
DYN3_MOUSE	393	97130	40	12	28	8	0.48	Dynamin-3	DNM3	Brain
AP2A2_MOUSE	321	103951	17	9	15	9	0.44	AP-2 complex subunit alpha-2	AP2A2	Kidney
ARF4_MOUSE	245	20384	15	8	8	6	3.18	ADP-ribosylation factor 4	ARF4	Kidney
ARF5_MOUSE	224	20517	19	11	9	6	3.15	ADP-ribosylation factor 5	ARF5	Kidney
ARF1_MOUSE	210	20684	13	10	9	7	3.11	ADP-ribosylation factor 1	ARF1	Brain
ARF6_MOUSE	172	20069	8	5	6	4	1.3	ADP-ribosylation factor 6	ARF6	Kidney
AP3B2_MOUSE	131	119118	20	4	19	4	0.15	AP-3 complex subunit beta-2	AP3B2	Brain
ARF4_MOUSE	120	20384	6	6	5	5	1.77	ADP-ribosylation factor 4	ARF4	Brain
VPS35_MOUSE	115	91655	6	2	5	1	0.05	Vacuolar protein sorting-associated protein 35	VPS35	Brain
AP1S1_MOUSE	115	18721	2	2	2	2	0.56	AP-1 complex subunit sigma-1A	AP1S1	Kidney
ARF6_MOUSE	113	20069	5	3	4	3	0.86	ADP-ribosylation factor 6	ARF6	Brain
CLH1_MOUSE	104	191435	12	4	7	4	0.09	Clathrin heavy chain 1	CLTC	Kidney

Supplementary Figure 4.

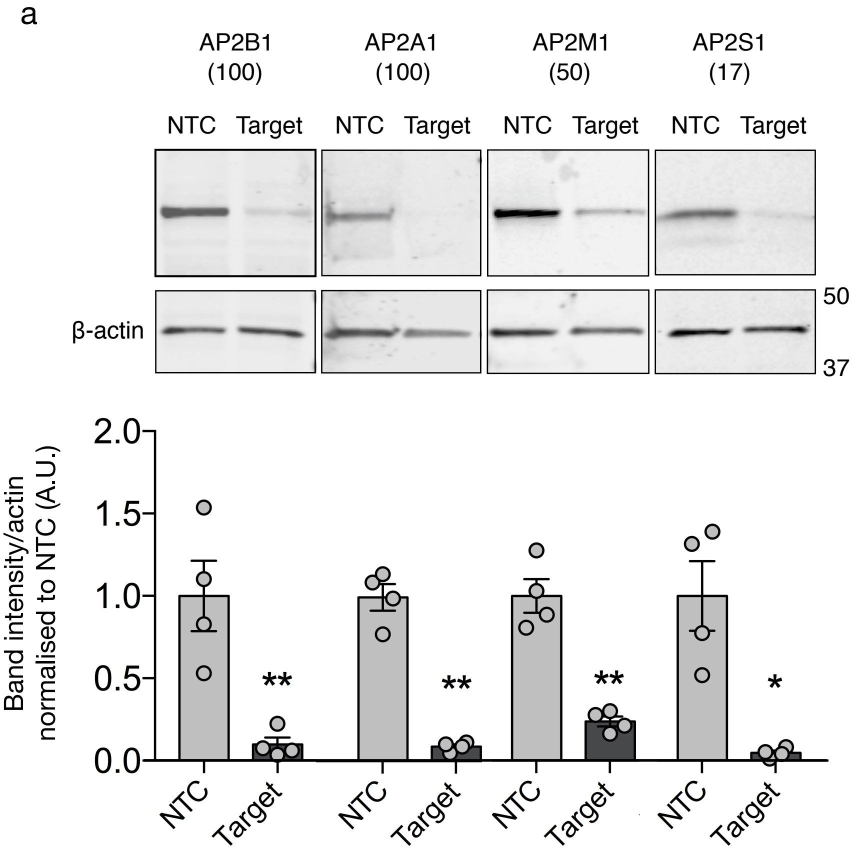

Supplementary Figure 5.

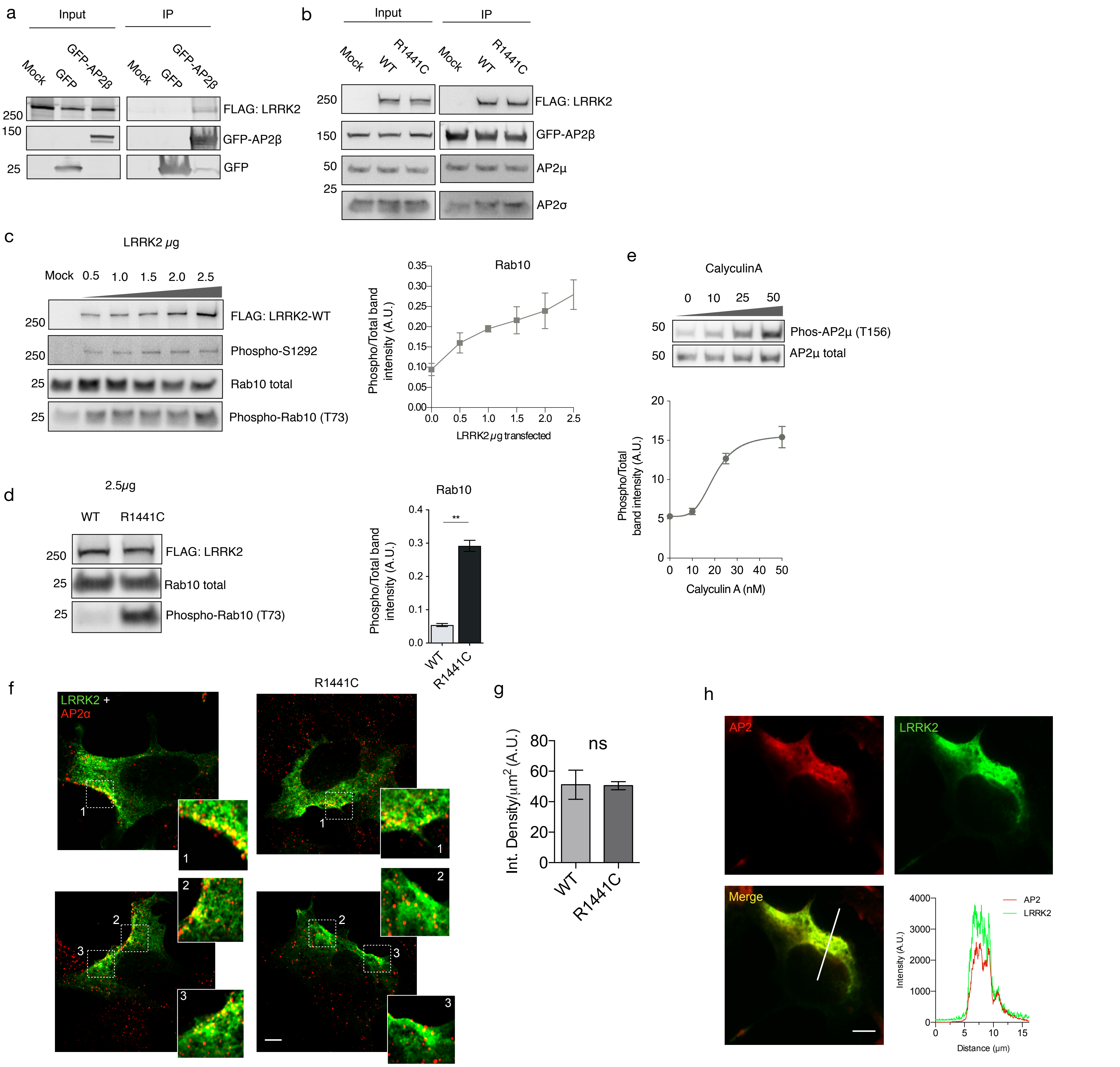

Supplementary Figure 6.

a

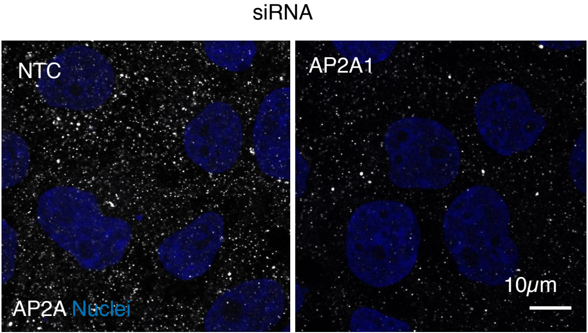

Supplementary figure 7.

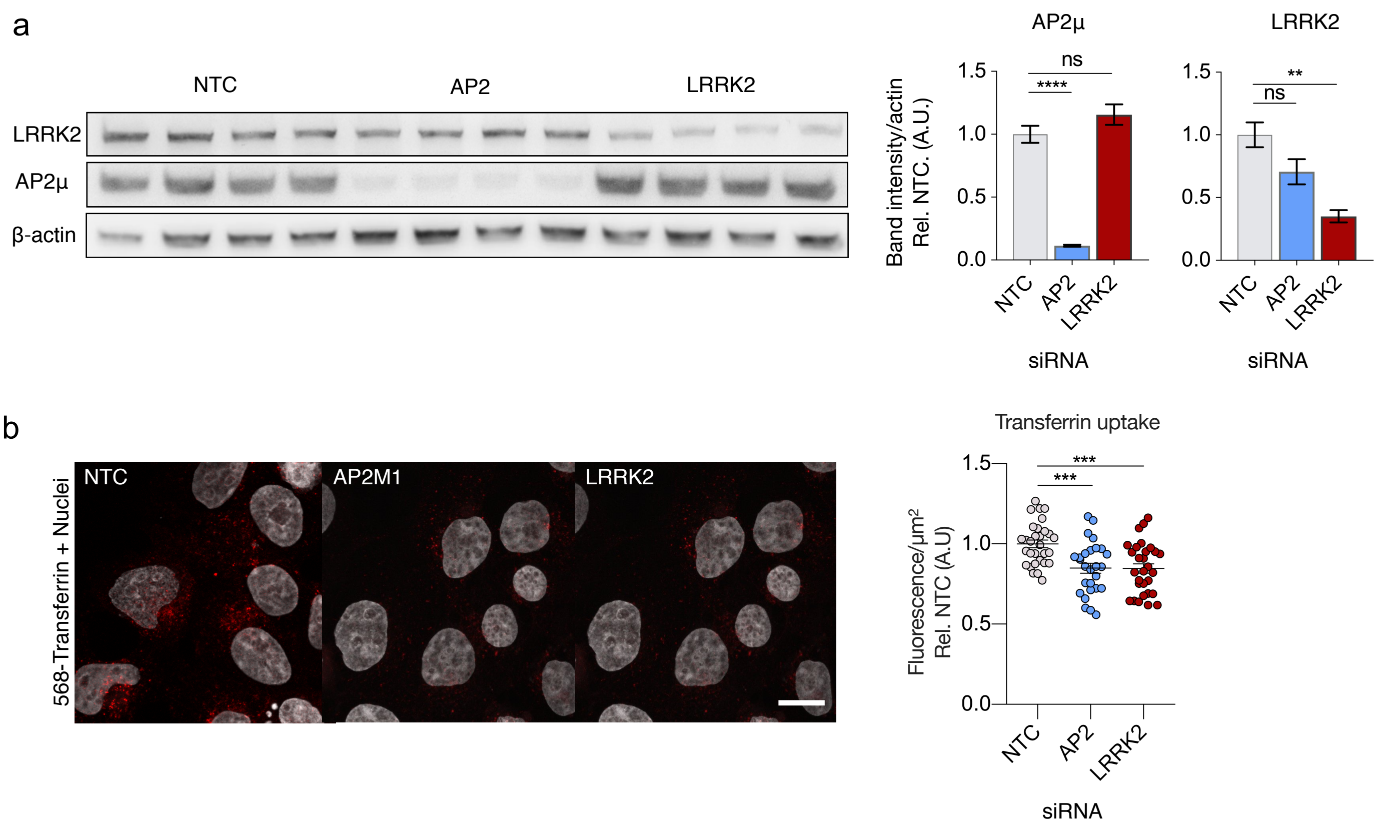

Supplementary figure 8.

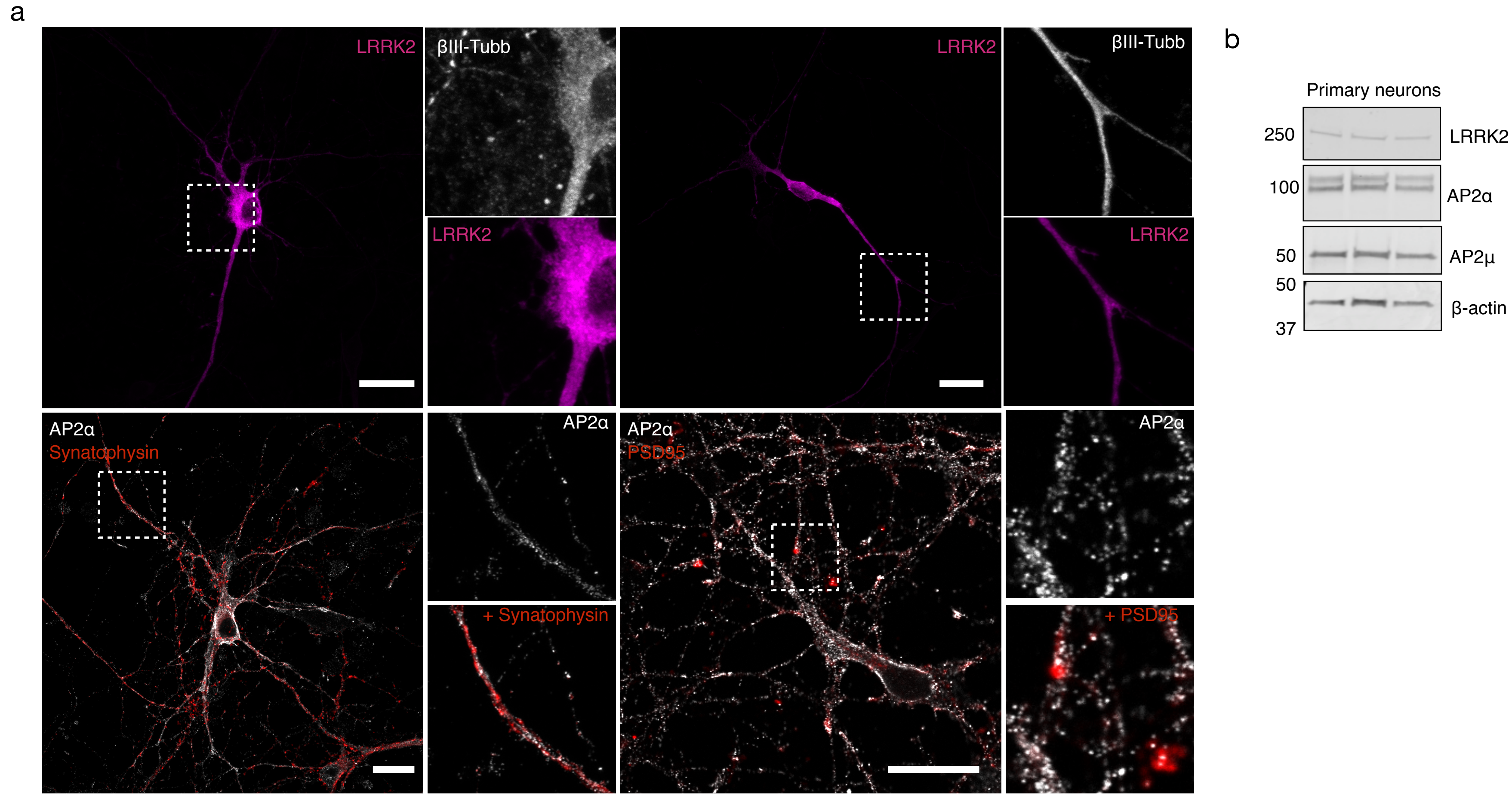

Supplementary table 9.

Study	Cases (N)	Controls (N)	Total (N)	Case age at onset (mean , SD in years)	Control age at last exam (mean, SD in years)
Baylor UMary Parkinson’s	789	195	984	64.9 (10.11)	65.45 (8.31)
Finnish Parkinson's	386	493	879	55.27 (5.64)	92.35 (3.86)
McGill Parkinson's	583	906	1489	65.71 (9.78)	55.79 (10.69)
Oslo Parkinson's Disease Study	476	462	938	50 (4)	61.85 (11.06)
Spanish Parkinson’s	1920	1164	3084	60.07 (12.70)	69.02 (9.95)
Vance (dbGap phs000394)	621	303	924	NA	81.88 (12.73)
Tubingen	741	944	1685	55.76 (11.55)	47.42 (12.38)
TOTAL	5516	4467	9983		
